# Modulating Whiteleg Shrimp (*Penaeus vannamei*) Health from the Inside out: Effects of Xylooligosaccharides from *Salicornia ramosissima* on Gut Metabolites and Microbial Community

**DOI:** 10.3390/ijms262411978

**Published:** 2025-12-12

**Authors:** Ana Garcia, Sergio Fernández-Boo, André Barreto, Miguel Semedo, Mette Hedegaard Thomsen, Allan Stensballe, Maxwel Monção, Leonidas Matsakas, Paul Christakopoulos, Viswanath Kiron, Rui J. M. Rocha, Benjamin Costas

**Affiliations:** 1 Interdisciplinary Centre of Marine and Environmental Research (CIIMAR), 4450-208 Matosinhos, Portugal; afgarcia@ciimar.up.pt (A.G.); msemedo@ciimar.up.pt (M.S.); 2 School of Medicine and Biomedical Sciences, University of Porto, 4050-313 Porto, Portugal; 3 RIASEARCH Lda., 3880-394 Murtosa, Portugal; andrebarreto@riasearch.pt (A.B.);; 4 Department of Chemistry and Bioscience, The Faculty of Engineering and Science, Aalborg University, 6700 Esbjerg, Denmark; 5 Department of Health Science and Technology, The Faculty of Medicine, Aalborg University, 9260 Gistrup, Denmark; 6 Biochemical Process Engineering, Division of Chemical Engineering, Department of Civil, Environmental and Natural Resources Engineering, Luleå University of Technology, SE-971 87 Luleå, Sweden; 7 Aquaculture Research Group, Faculty of Biosciences and Aquaculture, Nord University, 8049 Bodø, Norway

**Keywords:** *Penaeus vannamei*, halophytes, XOS, microbiota

## Abstract

Whiteleg shrimp (*Penaeus vannamei*) is currently facing significant challenges related to severe disease outbreaks. As the importance of the host–microbiota relationship is being revealed, modulating this relationship has become a key strategy in disease management. Xylooligosaccharides (XOS)—short-chain sugar molecules—have been gaining attention for their potential health benefits in the prebiotics field. In this study, an XOS-rich extract from *Salicornia ramosissima* was incorporated into shrimp feeds to evaluate its impact on gut health, with the main focus on gut proteomics and microbiota. XOS were incorporated at 0.1% (XOS_0.1) and 1% (XOS_1) concentrations, and a 14-day feeding trial, followed by a bacterial challenge with *Vibrio harveyi*, was performed. The effects of XOS were evaluated by assessing zootechnical parameters, gene expression in the hepatopancreas, and gut microbiota and proteome. The results showed no significant differences in zootechnical parameters and gene expression after the 14-day trial between animals fed XOS diets and control. However, shrimp fed XOS_1 showed an increased diversity of the microbial communities in the gut when compared with those fed control. Also, known shrimp gut symbionts, such as *Ruegeria*, *Leisingera*, and *Demequina*, were significantly enriched in groups fed XOS after the feeding trial. XOS also modulated the regulation of proteins in the gut. Nevertheless, stressful conditions appear to alter the effects of XOS and the dynamics of gut bacteria. Further studies are warranted to understand the impacts of long-term inclusion of XOS extracts, especially on health-related parameters and disease resistance.

## 1. Introduction

The aquaculture era has coincided with a time of environmental responsibility. Consumers’ awareness of sustainability and animal welfare is impacting the present and will shape the future of the animal feed industry. While most cultured aquatic species have been facing significant challenges from diseases, shrimp is experiencing particularly severe losses [1,2]. Frequent outbreaks of epidemics, mainly caused by Gram-negative bacteria such as *Vibrio* spp., *V. harveyi*, *V. vulnificus*, and *V. parahaemolyticus* and viruses such as white spot syndrome virus, have been increasingly impacting the shrimp industry [3,4,5,6]. A recent report from the World Aquaculture Society about the challenges of disease management in aquaculture highlights the potential benefits of employing functional feeds, such as immunostimulants and feed additives, as a preventive measure [2]. The influence of functional feeds on gut microbiota is a recent breakthrough, with promising applications for improvement of aquaculture species’ health [7].

The use of prebiotics as feed additives has been explored as a chemical and antibiotic-free way of improving health and fighting disease [8]. These complex carbohydrates, typically oligosaccharides, have two main ways of promoting an immune response: (i) serving as a fuel for probiotics in the gut and (ii) stimulating the host immune system [9,10]. Alongside many other health-promoting compounds, prebiotics are found in a variety of plant-based sources [11,12,13]. Xylooligosaccharides (XOS) are short-chain polymers derived from hemicellulose, one of the main structures of lignocellulosic biomass, through the depolymerization of xylan [14]. Recently, XOS were included in the list of candidates of accepted prebiotics of the International Association of Probiotics and Prebiotics (ISAPP) consensus statement on the definition and scope of prebiotics [15]. Therefore, in the last decade, several studies have focused on understanding the effects of XOS in growth performance, immunity, gut morphology, disease resistance, and overall influence on various aspects of animal health [16,17,18,19]. However, despite existing research on prebiotics in other contexts, to the best of our knowledge, no in vivo trials have specifically assessed the impact of XOS on the shrimp gut microbiota.

Halophytes can successfully grow and thrive in dry and salty environments, an important fact to consider in the current climate change scenario where desert areas continue to expand at an alarming rate [20]. In this context, drought and salt tolerant plants may play an important role in fighting desertification while providing food for a growing human population. The very traits that allow halophytes to succeed in arid conditions also equip them with a wealth of biologically active compounds [21]. Some of these plants, such as *Salicornia ramosissima*, are already used for human consumption, but the non-edible parts are often discarded while still holding an untapped potential [22]. A previous study by Monção et al. (2023) [23] demonstrated the possibility of using residual dejuiced fibers of S. *ramosissima* following a biorefinery approach. For instance, it was possible to yield hemicellulose fractions as well as an increased ratio of oligomers in samples. The main branched oligosaccharides were hexurono-XOS/alduronic acids. Lignin fractions had low sugar and ash contamination and low molecular weight—characteristics desired for further applications. Therefore, the present study aimed to investigate the effects of XOS derived from *S. ramosissima* non-edible fibers on shrimp growth performance, gut protein expression, microbiota composition, and immune modulation after exposure to the bacterial pathogen *V. harveyi*.

## 2. Results

### 2.1. Growth Performance and Survival

No significant differences were observed in final body weight, RGR, FCR, feed intake, and survival values between shrimp fed control or XOS diets (XOS_0.1 and XOS_1) after the 14-day feeding trial (0 h) (Table 1). Also, no mortality was observed during or after the bacterial challenge (72 h).

### 2.2. Hepatopancreas Gene Expression

Gene expression of immune- and stress-related genes was assessed in the hepatopancreas. No differences were found between dietary groups or time points (Table 2). Also, pathogen load, measured in the hepatopancreas, showed non-significant differences between challenged and non-challenged animals at the 72 h time point.

### 2.3. Microbial Composition of the Gut

**Figure 1:**
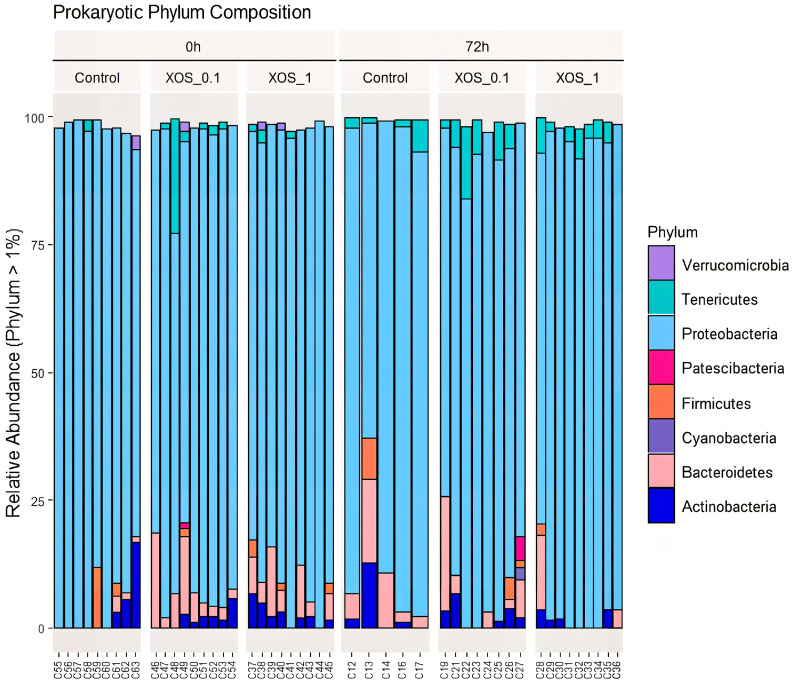
Bar plot of the relative abundance of the main phyla (abundance > 1%) observed in the analysis of the whiteleg shrimp gut microbiota. Samples are divided by dietary treatments (control, XOS_0.1, and XOS_1) and time points (0 h and 72 h after bacterial challenge). Different colors represent different phylum.

Individuals were grouped by time point and diet. A total number of 63 samples originated 552,913 high-quality sequences (Supplementary Data I). After removing samples with low sequence number (<10,000), a total of 55 samples were used for the downstream analysis, corresponding to a total of 2913 ASVs. *Proteobacteria* was undoubtedly the most abundant taxa (Figure 1), with relative abundance ranging from 62 to 99% of total sequences. In addition to *Proteobacteria*, *Actinobacteria* (1 to 22%) and *Bacteroidetes* (1 to 22%) were also prevalent across samples.

**Figure 2:**
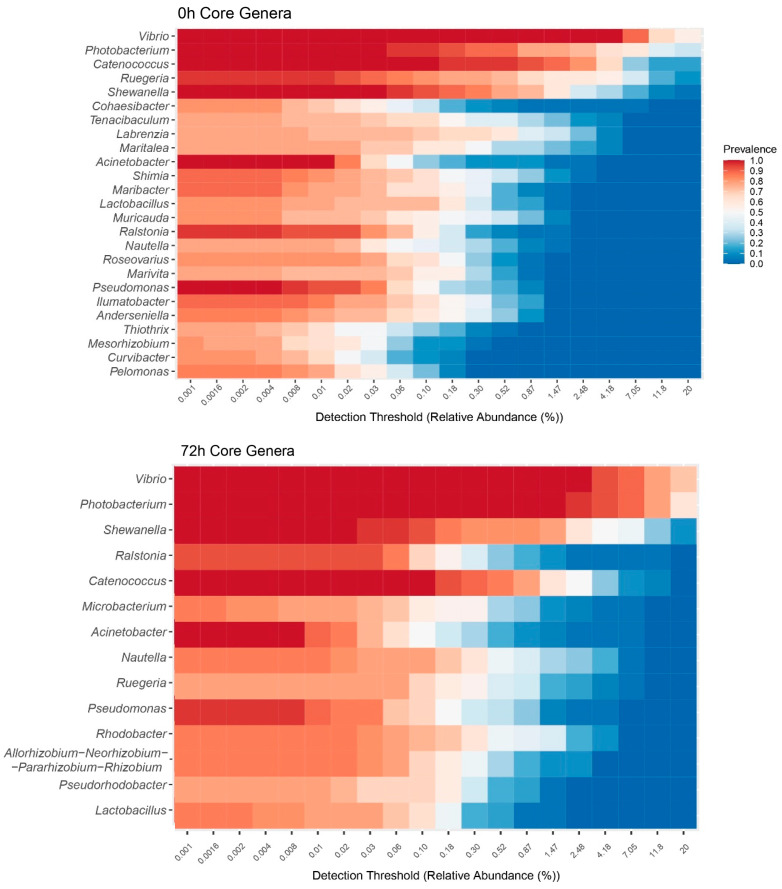
Heatmap figure of the relative abundance of the core bacterial genera in shrimp gut microbiota at 0 h (immediately after the feeding trial) and at 72 h (after the bacterial challenge). The color in each cell represents the mean relative abundance of that genus in the samples where it was detected above the corresponding threshold. The genera (rows) are sorted by their overall mean prevalence across all samples.

The most prevalent genera of *Proteobacteria* were *Vibrio* and *Photobacterium*, both from the *Gammaproteobacteria* class (Figure 2).

To identify the gut microbiota signature associated with the inclusion of XOS extracts, the abundance of each genus was compared among the different treatments and at the different time points: after the feeding trial (0 h) and after the bacterial challenge (72 h). Dietary groups and time points significantly influenced the abundance of diverse bacterial genera (Supplementary Data II). Shrimp fed XOS_0.1 and XOS_1 exhibited distinct microbiota profiles compared to the control group at both 0 h and 72 h post-challenge. Specifically, XOS_0.1-fed shrimp displayed a lower abundance of eight known genera and a higher abundance of eighteen genera at 0 h. At the same time point, shrimp fed XOS_1 diet showed a lower abundance of 13 genera and a higher abundance of 26 genera (Table 3).

At 72 h post-challenge, both XOS_0.1 and XOS_1 groups demonstrated notable shifts in microbiota composition. Inclusion of XOS_0.1 led to a lower abundance of 18 different genera and a higher abundance of 18 genera. Inclusion of XOS_1 resulted in a lower abundance of eighteen genera and a higher abundance of four genera (Table 3).

**Figure 3:**
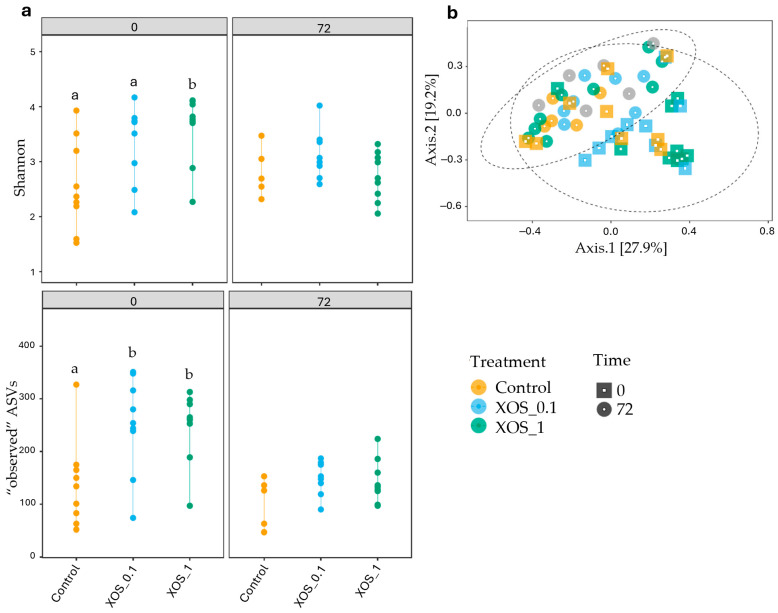
(**a**) Shannon and “observed” ASVs indexes of the different studied groups at 0 and 72 h, represented by different colors (yellow: control, blue: XOS_0.1, and green: XOS_1). Letters represent significant differences between groups. (**b**) Principal coordinates analysis (PCoA) graph performed by Bray–Curtis dissimilarities for all animals. Different colors represent different treatments (yellow: control, blue: XOS_0.1, and green: XOS_1), and different shapes represent different time points (square: 0 h and circle: 72 h) performed by Bray–Curtis dissimilarities representing the microbiome composition for all animals.

β-diversity analysis, as visualized through PCoA in Figure 3b, shed light on the principal sources of variation among samples. The PCoA plot clearly demonstrates the clustering of samples based on different time points. The PERMANOVA test confirmed the significance of time points as the primary driver of variation in microbial community composition, explaining 11.58% of the overall variation (*p* < 0.001). While diet contributed to a lesser extent, it was still a significant factor, explaining 7.6% of the overall variation (*p* < 0.05).

The XOS_1 diet significantly enhanced microbial alpha diversity compared to the control diet, as evidenced by increased Shannon and “observed” ASVs indices (*p* < 0.05) (Supplementary Data III and IV) (Figure 3a). Plus, “observed” ASVs indexes also indicated differences between XOS_0.1 and control at 0 h. However, these effects were diminished following exposure to a bacterial challenge. No significant differences in α-diversity were observed between infected and non-infected shrimp 72 h post-challenge regardless of dietary treatment.

### 2.4. Gut Proteome Profile

**Figure 4:**
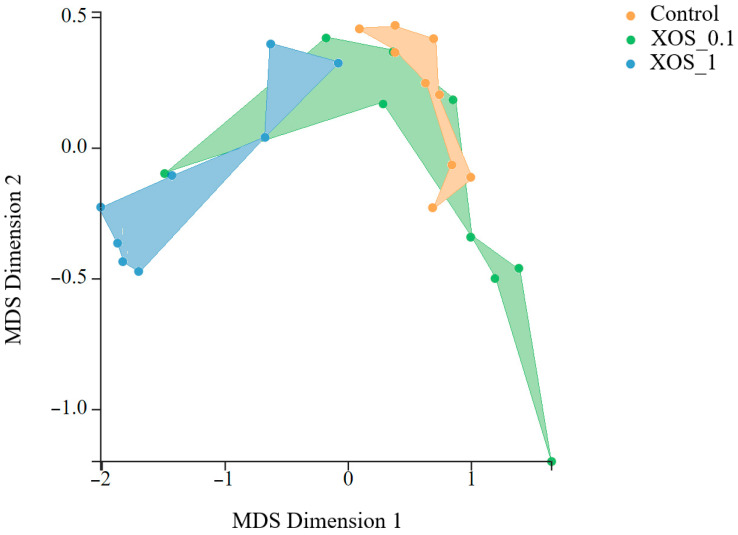
MDS analysis of shrimp gut proteome at 0 h using Degust software (available at https://degust.erc.monash.edu/, accessed on 16 September 2025). The plot represents the clustering of different dietary groups (control: orange, XOS_0.1: green, XOS_1: blue).

For proteomic studies on shrimp gut, nine animals per experimental condition were used. A total of 3348 proteins were used for statistical analysis after quality control. A log2 fold-change was applied to protein abundance values relative to the control. At 0 h, XOS groups showed a different gut proteome profile compared to control (Figure 4). Shrimp fed XOS_0.1 exhibited increased levels of 82 proteins and decreased levels of 54 proteins. At the same time point, shrimp fed XOS_1 displayed altered levels of 281 proteins compared to the control, with 40 proteins upregulated and 241 downregulated. A comparison of dietary groups after the bacterial challenge identified two upregulated and two downregulated proteins between control and XOS_0.1. No proteins were found differentially regulated between control and XOS_1. A total of 32 proteins were found to be differentially abundant between control groups before and after transport. Also, four proteins were differentially abundant between infected and non-infected control groups at the 72-h time point.

A functional enrichment analysis of differentially regulated proteins among dietary groups was performed using the DAVID database (Supplementary Data V). At 0 h, XOS_0.1 showed that the downregulated proteins are involved in metabolic pathways; protein synthesis; and SUMOylation, RNA degradation, proteolysis, oxidative phosphorylation, cytoskeleton, and lysosome pathways, among others. Upregulated proteins were related to metabolic pathways, glycan degradation, sphingolipid metabolism, proteolysis, lysosome pathways, etc. At the same time point, XOS_1 inclusion was responsible for a downregulation of proteins related to pathways that are involved in cellular activity related to amino acid metabolism, RNA polymerase, protein synthesis, processing and degradation, butanoate metabolism, glycolysis, oxidative phosphorylation, proteolysis, lysosome metabolism, and others. On the other hand, it led to an increase in cellular processes related to lysosomal activity, pyrimidine and retinol metabolism, autophagy, sphingolipid metabolism, motor and cytoskeleton proteins, etc. Functional analysis at 72 h post-infection showed that only the spliceosome pathway was downregulated with XOS_0.1 inclusion, and the RNA degradation pathway was upregulated in the same group. Comparison of non-infected animals fed control at 0 and 72 h showed a downregulation of RNA polymerase, nucleotide excision repair, motor proteins, cytoskeleton in muscle cells, and sphingolipid metabolism at 72 h. Upregulated proteins are linked to protein turnover, energy metabolism, antioxidant defense, and cellular adaptation pathways. Finally, challenged animals fed control diets showed a downregulation of sphingolipid metabolism compared to non-infected controls.

To identify densely connected groups of proteins within the protein–protein interactions (PPI) networks, MCL clustering was applied using the STRING database. For this analysis, the list of up- and downregulated proteins from shrimp fed XOS diets compared to control at 0 h was used. The main cluster found in the XOS_0.1 group was linked to RNA processing, transcription, protein modification, and metabolism. For the XOS_1 groups, the main cluster, containing 11 proteins, was related to mRNA transport, nuclear import, DNA replication, and protein synthesis. A second cluster, linked to the previous one, is focused on cytoplasmic translational initiation and translation initiation factor activity. Both clusters comprised downregulated proteins. Upregulated proteins were grouped together in clusters related to (i) innate immunity and lipid metabolism and (ii) peptidase family. At 72 h, no significant clusters were identified in either XOS groups or between infected and non-infected groups. Finally, MCL clustering analysis of the differentially abundant proteins from shrimp fed the control diet before and after transport revealed a single cluster involving two proteins related to carbohydrate and amino acid metabolism.

## 3. Discussion

As the first in vivo assessment of XOS extract from *S. ramosissima* in aquafeeds for shrimp, this short-term trial was designed to screen the primary effects of XOS. The main aim was to identify its effects on shrimp gut microbiota, complemented by an analysis of gut proteome and of key immune-related genes from the hepatopancreas. The short duration of this trial was strategically designed, not only for resource efficiency but also from a biological perspective: since XOS is hypothesized to possess immunostimulatory roles, long-term immune response could lead to the exhaustion of the host defense system [24]. The inclusion of *S*. *ramosissima* XOS extracts (either XOS_0.1 and XOS_1) did not result in significant changes in zootechnical parameters. A study by Sun et al. (2019) [25] investigated the effects of XOS on the growth performance of whiteleg shrimp for 60 days. The authors reported significant improvements in feed efficiency and survival rate in animals fed XOS diets at inclusion levels of 0.4 to 0.6% compared with control. Nonetheless, XOS inclusion levels of 0.1 to 0.6% did not have an impact on growth and chemical composition of shrimp. In the present study, FCR showed no differences among control- and XOS-fed groups. While longer periods of supplementation with prebiotics are not always associated with improved effects on growth, XOS-enriched fractions could be considered for longer feeding trials, with the aim to study growth performance and feed efficiency in shrimp [26]. Gene expression analysis also showed no differences between dietary groups. However, it is worth noting the presence of high standard errors for some of the analyzed genes, suggesting high biological variability within the same group. While the causes are not fully understood, this variability is likely due to several biological factors common in studies with crustaceans [27,28]. Since the molting stage was not considered in the present study, individuals at different points of this cycle will likely have different gene expression profiles. Nonetheless, the hepatopancreas is a complex organ, serving roles such as digestion, detoxification, and immune response [29]. As such, the effects related to the XOS treatments may have been masked by the background noise associated with these physiological processes. To better understand the underlying mechanisms of XOS effects, a deeper analysis on shrimp gut microbiota was performed. Like other prebiotics, XOS selectively stimulates the growth of beneficial bacteria in the gut [30]. This promotes a healthy environment by competing with pathogens for resources and space and by producing metabolites that modulate the host’s immune response [7]. In the present study, 16S rRNA gene sequencing revealed a dose-dependent effect of XOS on the composition of microbial communities in the shrimp gut. While the knowledge of the interactions between bacteria and shrimp is still uncharted, the potential roles of certain bacteria have been addressed in the whiteleg shrimp. In this study, *Demequina*, *Leisingera*, *Paracoccus*, *Ruegeria*, and *Tamlana* are among the genera found more abundant in shrimp fed both XOS_0.1 and XOS_1 diets at 0 h compared to control. These taxa have been associated with positive outcomes in shrimp health [31,32,33,34,35,36,37]. Among the identified genera, *Ruegeria* and *Leisingera* are known to exhibit antagonist effects against several pathogenic bacteria and to improve shrimp immunity [31,32]. *Flavobacterium*, found to be more abundant in shrimp fed XOS diets, has been historically associated with fish diseases. However, recent studies suggest that *Flavobacterium* is not pathogenic to shrimp and seems to be part of the core microbial community of shrimp [34]. In a recent study, Fu et al. (2024) [36] identified a group of bacteria that included *Paracoccus* as crucial for maintaining host fitness by stabilizing the gut bacterial community. The same study identified operational taxonomic units (OTUs) belonging to *Tenacibaculum*, *Xanthomarina*, and *Demequina* as potentially involved in disease suppression. In the present study, all these bacteria were more abundant at 0 h in shrimp fed the XOS diet compared to control. Nonetheless, the role of *Tenacibaculum* in shrimp health remains complex, as different species from this genus have been found in both healthy and diseased shrimp [37,38]. *Muricauda*, *Gilvimarinus*, and *Luteolibacter* were also consistently more abundant in shrimp fed XOS diets at 0 h, with no clear role on shrimp immunity [37,39,40]. However, at 0 h, *Fusibacter*, a genus associated with improved antioxidant capacity and immune enzyme activity in shrimp, was less abundant in shrimp fed XOS diets [41]. Similarly, *Acinetobacter*, recently linked to mortalities in aquaculture and a potential pathogen of red leg disease in whiteleg shrimp, was also less abundant under the same conditions [42,43]. Notably, shrimp fed a higher inclusion of XOS showed a higher abundance of *Bacillus* compared to control at 0 h. *Bacillus* is one of the most used probiotic bacteria in fish and has been shown to decrease shrimp mortality related to acute hepatopancreatic necrosis disease (AHPND) [44,45]. However, the effects of the XOS_1 diet on *Bacillus* abundance were no longer visible after challenge. In fact, the same happened with most of the previously mentioned bacteria. Indeed, only four bacterial genera were more abundant in challenged shrimp fed the XOS_1 diet compared to control at 72 h. Among these bacteria, only *Weissella*, a lactic acid bacterium, has been studied for its role in shrimp health, with a strong inhibitory effect against *V. parahaemolyticus* causing AHPND [46]. Contradictory outcomes from different studies investigating the same bacterial taxa make extrapolation difficult and, as so, also challenging to draw solid conclusions. This highlights the necessity to expand our knowledge on the specific functions of bacteria within the shrimp gut and their impact on overall shrimp health.

In par with bacterial composition, diversity is also a key player on ecosystem stability and resilience [47]. While composition refers strictly to the taxonomic identity of the bacteria present, diversity (e.g., the Shannon index) is a more complex measure as it considers both richness (the number of different bacterial types) and evenness (how equally distributed those types are). Microbiota studies have consistently linked microbial diversity to overall health in various organisms [48,49,50]. Research on shrimp exposed to *V. harveyi* has shown a decrease in gut microbial diversity following infection, suggesting a potential correlation between diversity and disease resistance [6]. Here, the highest inclusion of XOS was responsible for increased community richness and evenness after the feeding trial (0 h), as indicated by the Shannon index mean values (3.604) compared to control (2.569). This is revealing of a more diverse and balanced gut microbiota. While this outcome evidences the dose-dependent effect of XOS extract, future studies should address chemical analysis to evaluate the stability of XOS during storage and immersion, confirming the exact dosage of XOS delivered to shrimp. The lack of significant differences on α-diversity indexes, before and after challenge and between infected and non-infected animals, indicates that neither the transport nor the bacterial challenge changed the diversity of the gut microbial community. Interestingly, these findings are in agreement with other studies addressing *Vibrio* infection in shrimp, where authors did not find differences in bacterial community diversity in early stages of the disease [51,52]. Nonetheless, the authors observed a decrease in bacterial diversity during the disease progression, which was not evaluated in our short-term study. The evaluation of pathogen load in the hepatopancreas and gut showed no differences before and after challenge. In fact, studies investigating *Vibrio* kinetics showed that the peak usually occurs 12 h after exposure, and the pathogen is often clear at 72 h post-challenge [53]. Nonetheless, while the pathogen was not detected, it is possible that the toxins outlast its presence. Studies investigating *Vibrio* infection in shrimp, in particularly hepatopancreas lesions, suggested that the pathologies are caused by secreted toxins from the bacteria rather than the presence of the bacteria itself [53,54].

Overall, microbiota results indicate that XOS induced a more diverse microbial community. In addition, it promoted the proliferation of some bacteria (such as *Ruegeria* and *Leisingera*), previously reported as being positively correlated with the gut microbiota of healthy shrimp. However, it is likely that, when included at the tested concentrations, the beneficial effects of XOS may fade under stress conditions, as observed in our bacterial challenge. Nonetheless, this loss of effect after the challenge highlights that subsequent studies must incorporate an extended bacterial challenge to evaluate the impact of XOS extracts on mortality, disease resistance, and recovery capacity.

Proteomic studies may help us to delve deeper into the effects of XOS and the interplay between stress and diet. Interestingly, contrary to the microbiota findings, the proteomic profile remained largely unaffected by the bacterial challenge.

Regarding shrimp fed XOS_0.1, changes in carbohydrate metabolism and energy production pathways suggest an alteration of metabolic processes. This could be linked to a change in energy demand, the presence of new metabolites, or even cellular stress. The downregulation of protein synthesis and degradation pathways seems to point to a shift in host metabolic activity [55,56]. The consistent presence of the lysosomal pathway as being both up- and downregulated suggests a complex role for lysosomes in response to the XOS_0.1 diet. PPI network revealed that the main cluster might represent a multifunctional complex involved in various cellular processes. The combination of transcription, RNA processing, protein modification, and energy metabolism indicates that the cluster is functionally diverse and is likely involved in gene expression regulation and metabolic processes [56]. A higher dietary inclusion of XOS, like was previously seen in XOS_0.1, was responsible for both up- and downregulation of lysosome pathways. Also, the downregulation of oxidative phosphorylation, glycolysis, and the TCA cycle and upregulation of other metabolic pathways, such as pyrimidine metabolism and retinol metabolism, may point to a change in host metabolic priorities. The downregulation of proteasome and ubiquitin-mediated proteolysis could indicate a decrease in protein breakdown. However, the upregulation of autophagy complicates this picture. Both proteolysis and autophagy are involved in nutrient recycling [56]. PPI network analysis identified two primary clusters of downregulated proteins linked together. One of the clusters was significantly enriched in proteins related to gene expression and cellular regulation, while the other was associated with protein synthesis. Conversely, distinct clusters of upregulated proteins emerged, encompassing functions in innate immunity, lipid metabolism, and peptidase activity. The interplay between upregulated and downregulated proteins is intriguing as it suggests a complex regulatory response. Although XOS might have direct interactions with host cells, they may also indirectly influence cellular processes by modulating the bacteria in the shrimp gut. While changes in dietary fibers alter the composition of gut microbiota, microbiota itself can act as a metabolic organ [57,58]. Indeed, it is known that gut microbial composition modulates the production of metabolites, such as short-chain fatty acids and vitamins [55]. This way, XOS may be exerting its influence on host physiology by regulating metabolic processes, protein expression, and immune responses, among others. Nonetheless, while the 14-day period highlighted the potential of XOS on modulating shrimp gut microbiota, a longer trial would be important for the detection of putative long-term effects in shrimp health.

## 4. Materials and Methods

### 4.1. Salicornia Ramosissima Biomass and Xylooligosaccharides Production

*S. ramosissima* biomass was harvested in Murtosa (Portugal) after 26 weeks of cultivation in sandy soil and irrigation with water containing 12–13 g L^−1^ NaCl, as described by Monção et al. (2023) [23]. Biomass was dejuiced with a lab-scale single-auger juicer (Omega EUJ-707, Sana, Ceske Budejovice, Czech Republic), yielding 66.7% juice and 33.3% fibers. Untreated dejuiced fibers included 16.64% *w*/*w* cellulose, 22.42% *w*/*w* hemicelluloses, 15.86% *w*/*w* lignin, 32.53% *w*/*w* extractives, and 0.78% *w*/*w* ashes (total ashes including those in the extractives amounted to 13.21%) [23]. The fibers were fractionated in an organosolv reactor (Haato, Vantaa, Finland) at 200 °C for 45 min with 60% *v*/*v* ethanol content and a liquid-to-solid ratio of 10 (L/kg), as previously described [23]. After organosolv, the slurry was vacuum filtered to separate the cellulose-riched insoluble pulps from the process liquor. This liquor was processed in a vacuum evaporator to remove the ethanol, followed by centrifugation to precipitate lignin. The remaining aqueous solution containing the dissolved hemicellulose fraction was collected, freeze-dried, and stored at room temperature before being used in the shrimp feed trials. The resulting powder contained 7.7% oligomeric sugars (xylan, 4.63%; mannan, 1.63%; glucan, 1.11%; rhamnan, 0.33%), 3.98% monosaccharides (arabinose, 1.88%; xylose, 1.39%; mannose, 0.51%; glucose, 0.14%; rhamnose, 0.06%), and 0.34% uronic acids (glucuronic acid, 0.32%; galacturonic acid, 0.02%) [23].

### 4.2. Dietary Treatment

Three experimental diets were formulated for juvenile whiteleg shrimp (*Penaeus vannamei*): a control diet (CTRL) designed to meet their standard nutritional requirements and two test diets based on the CTRL formulation but supplemented with *S. ramosissima* XOS. The XOS-enriched diets contained 0.1% (XOS_0.1) and 1% (XOS_1) XOS, respectively (Table 4). The proximate composition of all diets is presented in Table 5.

Diets were manufactured by SPAROS Lda. (Olhão, Portugal). All powder ingredients were mixed accordingly to the target formulation in a double-helix mixer (model RM90, MAINCA, Granollers, Spain) and ground (below 200 µm) in a micropulverizer hammer mill (model SH1, Hosokawa-Alpine, Augsburg, Germany). Subsequently, the oils were added to the mixtures, which were humidified with 20–25% water and agglomerated by a low-shear and low-temperature extrusion process (ITALPLAST, Trento, Italy). Extruded pellets (1.5 mm) were dried in a vibrating fluid bed dryer (model DR100, TGC Extrusion, Roullet-Saint-Estèphe, France). Diets were packed in sealed plastic buckets and shipped to the research site where they were stored at room temperature but in a fresh and aerated emplacement.

### 4.3. Experiment Design and Animal Sampling

**Figure 5:**
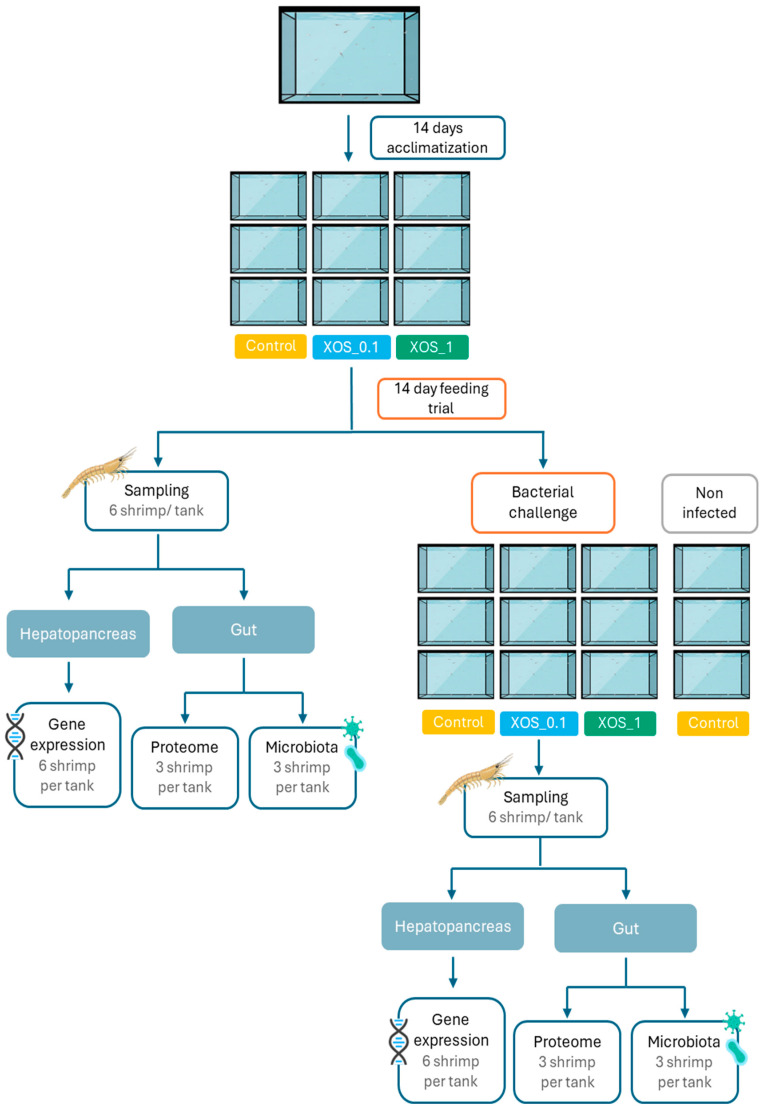
Representation of the experimental design of the 14-day feeding trial with whiteleg shrimp, followed by a bacterial challenge with *V. harveyi*.

Whiteleg shrimp juveniles (0.85 ± 0.01 g) were obtained from Miami Aquaculture (Boynton Beach, FL, USA) and transported to the RIASEARCH facilities (Murtosa, Portugal). Upon arrival, individuals were randomly distributed into 40 L tanks, part of a 4 m^3^ clear water recirculating system (RAS), housing 52 shrimp each, and maintained in acclimation for 14 days before the experiment (see Figure 5 for the experimental design scheme). Throughout the experiment, temperature was maintained at 27.9 ± 1.2 °C, dissolved oxygen was maintained at 6.6 ± 0.4 mg L^−1^, salinity was maintained at 20.6 ± 0.4 g L^−1^, pH was maintained at 7.4 ± 0.1, NH_3_ was maintained at 0.0 ± 0.0 mg L^−1^, and NO_2_ was maintained at 0.2 ± 0.1 mg L^−1^. Shrimp were kept under a 14 h light/10 h dark photoperiod and given 8 meals per day using automatic feeders. Each of the three dietary treatments was randomly assigned to three replicate tanks. Shrimp were fed ad libitum every day, 8 times a day, and the amount of feed was adjusted daily according to wasted feed. Mortality was monitored and recorded daily throughout the experiment. Following 14 days of feeding, after an overnight fasting period, shrimp were weighed and counted to determine mean body weight, relative growth rate (RGR), feed intake, feed conversion ratio (FCR), and survival. Six shrimp per tank (18 shrimp/dietary treatment) were randomly selected and euthanized in ice-cold water for tissue collection. The hepatopancreas of all sampled animals (n = 18) was collected and preserved in RNAlater solution for subsequent gene expression analysis. From the collected shrimp, the intestines of 3 shrimp per tank (n = 9) were used for microbiota evaluation, and the other 3 were used for proteome studies (n = 9), following aseptic procedures.

### 4.4. Bacterial Challenge with Vibrio Harveyi

Following the feeding trial, 40 shrimp per diet were transported to CIIMAR facilities (Matosinhos, Portugal) and distributed among 12 tanks (3 tanks per dietary treatment plus 3 additional tanks serving as control of infection). A 24-h acclimatization period allowed the shrimp to adjust to the new environment before the bacterial challenge.

*Vibrio harveyi* was cultured for 24 h at 25 °C in Triptic Soy Agar (TSA) supplemented with 2% NaCl. Following this initial growth phase, the bacteria were transferred to two Erlenmeyer flasks containing 400 mL of Triptic Soy Broth (TSB) supplemented with 2% NaCl and incubated at 25 °C with shaking at 140 rpm for another 24 h. To achieve the desired bacterial concentration, the culture’s optical density (OD) was measured and adjusted to a final concentration of 1 × 10^8^ colony forming units (cfu) mL^−1^. This concentration was subsequently confirmed using the drop plate (DP) method for cfu quantification [59]. A total of 9 shrimp per tank (3 tanks per condition) were collected and subjected to a 2-h bath infection in separated tanks, with each tank receiving 50 mL of the prepared *V. harveyi* suspension diluted in 10 L of water, leading to a final concentration of 1 × 10^6^ cfu mL^−1^. Also, 3 additional tanks of shrimp fed the control diet underwent a similar bath but using sterile TSB without bacteria (non-infected group), serving as a control to confirm the effectiveness of the challenge. Subsequently, shrimp were transferred back to their original tanks for recovery. Tissue collection occurred 72 h post-challenge, with six shrimp randomly sampled from each tank. Tissues were sampled as described in Section 4.3, with the hepatopancreas collected from 6 shrimp from each tank (n = 18) and preserved in RNAlater for gene expression analysis. The gut was aseptically dissected from 6 shrimp from each tank, 3 for microbiota (n = 9) and 3 for proteome (n = 9) studies, and flash-frozen in liquid nitrogen.

### 4.5. Production Efficiency and Growth Performance

RGR (% weight day^−1^) was calculated as RGR = (eg − 1) × 100, where g = (lnWf − lnWi) × t − 1. Wf and Wi correspond to the final and initial weights, respectively, and t is time. Feed conversion ratio (FCR) was calculated as FCR = (Fi/Wg), where Fi corresponds to feed intake (g) and Wg corresponds to the mean weight gain (g). Economic conversion ratio (ECR) was calculated as ECR = ((Fi × C)/Wg), where Fi corresponds to feed intake (Kg), C is diet price in € Kg^−1^, and Wg is the mean weight gain (Kg). Survival was expressed as percentage and calculated as S = (Sf/Si) × 100, where Si and Sf correspond to the initial and final number of individuals in the tanks, respectively.

### 4.6. Gene Expression Analysis

Total RNA from the hepatopancreas was extracted with the NZY total RNA isolation kit (NZYTech, Lisbon, Portugal), following the manufacturer’s protocol, with some modifications. Briefly, the entire hepatopancreas from each sample was transferred from the RNAlater to a 2 mL tube loaded with 1 mL of buffer NR and 10 µL of β-mercaptoethanol. Then, it was homogenized using the precellys 24 tissues homogenizer (BertinIns., Montigny-le-Bretonneux, France) by 2 cycles of 6000× *g* for 20 s. After that, 350 µL of the homogenate was applied into a NZYSpin Homogenization column and centrifuged for 1 min at 11,000× *g*. The following steps were completed as recommended by the manufacturer. The quantification and purity of RNA were estimated by spectrophotometry (DeNovix Inc., Wilmington, DE, USA), and RNA integrity was confirmed through a 1.5% agarose gel electrophoresis. Reverse transcription of the RNA was performed using the NZY First-Strand cDNA Synthesis Kit (NZYTech, Lisbon, Portugal), following the manufacturer’s protocol. Quantitative PCR (qPCR) assays were performed with 4 μL of cDNA (40 ng), with 5 μL of iQ SYBR green 2 × Supermix (Bio-Rad, Hercules, CA, USA) and 0.5 μL (10 mM) of forward and reverse primers, to a final volume of 10 μL. For the qPCR reaction, the following conditions were settled: a denaturation step of 95 °C for 10 min, followed by 40 cycles of 95 °C for 15 s, 60 °C for 30 s, followed by a melting curve from 60 to 95 °C, with increments of 0.5 °C for each 0.5 s, and finally a cycle of 95 °C for 15 s. The primers selected for this analysis are described in Table 6. RPL-8 and GADPH were selected as housekeeping genes, using the geometric mean of their expressions for the normalization of the expression of the target genes. For the detection and quantification of *V. harveyi*, specific primers targeting *mreB*, *topA*, and *toxR* genes were used, using the following conditions: 5 min at 95 °C, followed by 30 cycles of 1 min at 95 °C, 2 min 15 s at 55 °C, and 1 min 15 s at 72 °C, and an additional step of 7 min of extension at 72 °C.

### 4.7. Microbiota Studies

#### 4.7.1. gDNA Extraction and Preparation of 16S Sequencing

Genomic DNA was extracted from total gut samples using the DNeasy Blood & Tissue kit (QIAGEN, Hilden, Germany), according to the manufacturer instructions, with some modifications. A total of 9 shrimp per condition (3 per tank) were analyzed separately. First, samples were treated with 300 µL of an enzymatic lysis buffer containing 20 mM TrisCl, pH 8, 2 mM Sodium EDTA, 1.2% Triton X100, and 20 mg mL^−1^ lysozyme and incubated at 37 °C for 1 h. Then, 180 µL of this mixture was transferred to a new 1.5 mL tube, and 25 µL of proteinase K and 200 µL of buffer were added, followed by another incubation, this time at 56 °C for 1 h. After incubation, 200 µL of ethanol was added to the mixture and vortexed. The following steps were performed as described by the manufacturer in “Purification of total DNA from animal tissues (Spin-column protocol)”. DNA quantity and quality were determined by spectrophotometry (DeNovix Inc., Wilmington, DE, USA). PCR was performed using universal 16S rRNA gene bacterial primers to amplify V3–V4 regions: 341F (5′CCTACGGGNGGCWGCAG3′) and 805R (5′GACTACNVGGGTWTCTAATCC3′) [60]. For the PCR reaction, 2 µL of gDNA was added to a 25 µL mix containing 12 µL of NZYTaq II 2× Green Master Mix (NZYTech, Lisbon, Portugal) solution, 1 µL of each primer, and 8 µL of water. PCR conditions consisted of an initial denaturation step at 95 °C for 10 min (1 cycle), 35 cycles at 95 °C for 1 min, 50 °C for 45 s, 72 °C for 1 min, and a final extension step at 72 °C for 7 min. The presence of bacterial DNA was confirmed on a 1.5% agarose gel electrophoresis. A second PCR amplification of targeted regions was performed to connect the targeted regions with barcodes. PCR products from each sample were selected by a 2% agarose gel electrophoresis and pooled, end-paired, A-tailed, and further ligated with illumine adapters. Libraries were sequenced at Novogene (Novogene (UK) Company Limited, Cambridge, UK) on a paired-end Illumina platform to generate 250 bp paired-end raw reads. The library was checked with Qubit and real-time PCR for quantification and bioanalyzer for size distribution detection. Paired-end reads were assigned to samples based on their unique barcode and truncated by cutting of the barcode and primer sequence.

#### 4.7.2. Analysis of 16S Sequencing

High-throughput sequencing data obtained in FASTQ format underwent comprehensive analysis using the R software (version 4.4.1, R core team, Vienna, Austria). The upstream analysis was performed resorting to the DADA2 package (version 1.16). After the barcodes/adapters had been removed, the read quality profiles were checked, and reads were filtered using standard filtering parameters: maxN = 0 (DADA2 requires no Ns), truncQ = 2, rm.phix = TRUE and maxEE = 2. Forward and reverse sequences were merged, and after chimeras had been removed, amplicon sequence variants (ASVs) were assigned to taxonomy using the SILVA database (version 132). Prior to downstream analyses, stringent filtering was applied to remove samples with a low number of reads (<10,000), alongside non-bacterial sequences (eukaryotic, chloroplast, and mitochondria). To visualize bacterial community composition across taxonomic levels (phylum, class, genus), bar plots were constructed using the ggplot2 package from R software (version 4.4.1, R core team, Vienna, Austria). β-diversity, reflecting dissimilarities between samples, was assessed using the Bray–Curtis dissimilarity index and visualized through principal coordinate analysis (PCoA). α-diversity, capturing species richness and evenness within each sample, was estimated with the Shannon index. Species richness was estimated with the “observed” ASVs (total number of unique ASVs) method. A core microbiome heatmap was generated using “plot_core” from the vegan package from R software (version 4.4.1, R core team, Vienna, Austria) to visualize the prevalence of abundant genera detected at different time points.

### 4.8. Proteome Gut Profile

For quantitative proteomic analysis, 9 intestinal samples per dietary treatment were collected at 14 days (after the feeding trial) and at 72 h post-challenge (total of 63 samples). Sample processing adhered to the uSP3 protocol defined by [61]. Briefly, intestines were homogenized in lysis buffer (1% SDS, 100 mM AmBic, 10 mM TCEP, 40 mM 2-CAA) using bead beating (2 × 5 min at speed 5; Bullet blender gold, Next advance, Troy, NY, USA; SS 0.9–2.0 mm beads), followed by heat denaturation at 95 °C for 5 min. PureCube Carboxy MagBeads (Cubebiotec, Monheim am Rhein, Germany) were added at a 1:10 bead-to-protein ratio, and after magnetic separation and supernatant removal, samples were digested overnight at 37 °C with trypsin (Pierce, MS grade, Thermo Fisher Scientific Inc., Rockford, IL, USA). Digested peptides were then dried and resuspended in loading buffer (2% Acetonitrile, 0.1% FA). The concentrations were determined via fluorometry (Pierce™ Quantitative Peptide Assay, Thermo Fisher Scientific Inc., Rockford, IL, USA). Subsequently, 1 µg of peptides per replicate was subjected to LC–MS analysis on a ThermoSci Q-exactive HF-X Orbitrap instrument (ThermoSci, Bremen, Germany) in DIA mode. The chromatographic separation was performed on an IonOptiks Aurora Ultimate 25 × 75 C18 UHPLC column (IonOpticks Pty Ltd., Victoria, Australia) at 60 °C and a flow rate of 400 nL/min, employing a 90-min gradient within a runtime of 2 h.

### 4.9. Statistical Analysis

Differences in growth performance, feeding efficiency, and survival between dietary treatments were evaluated using one-way ANOVA, followed by Tukey HSD multiple comparison tests. Kruskal–Wallis one-way analysis of variance tests, followed by Wilcoxon pairwise comparison tests, were used when data did not comply with normality. In results expressed as percentage, an arcsine transformation was performed prior to any statistical test: T = ASIN (SQRT (value/100)). A two-way ANOVA was used to analyze differences in expression of the target genes, treating both time and treatment as fixed effects, using the SPSS Statistics 26 software package for Windows (IBM^®^ SPSS^®^ Statistics, New York, NY, USA). Results were expressed as means ± standard deviation (SD).

Regarding the gut microbiota data, data analysis was carried out using the R software, version 4.4.1 (R core team, Vienna, Austria). Significant differences in ASV abundance between different diets and time points were determined using DESeq2 package from R software (version 4.4.1, R core team, Vienna, Austria). Differential expression analysis was conducted with a Wald test and a parametric model. To define an ASV as differentially abundant, the following threshold was applied: the false discovery rate (FDR), using the Benjamini–Hochberg procedure, was set at <0.05, and the fold change (FC) was set at >2. A two-way ANOVA test was used find differences in alpha diversity, followed by Tukey’s HSD. The PERMANOVA was applied on beta diversity parameters. For proteomic analysis, differential protein expression analysis was performed using the Degust software (available at https://degust.erc.monash.edu/, accessed on 16 September 2025) [62]. Differentially expressed proteins were identified using an FDR threshold of 0.05 and an FC > 2.0, relative to the control group. Functional enrichment analysis (GO terms and KEGG pathways) was conducted using the DAVID database [63]. MCL clustering was applied using the STRING database [64].

## 5. Conclusions

The main goals of this study were i) to use the *S*. *ramosissima* by-products to generate a product with added value and ii) to understand the effects of the dietary inclusion of XOS on shrimp gut heath. Both gut microbiota and proteome results offered valuable outputs for this discussion. Under undisturbed conditions, dietary XOS appear to have a positive effect on shrimp gut microbiota diversity (XOS_1) and composition. XOS also seem to influence the regulation of different proteins in the shrimp gut, but further investigation is needed to fully understand their functional implications. However, when shrimp cope with a stress condition, such as a bacterial bath challenge, there is an attenuation of dietary effects on the gut proteomic profile. Nonetheless, even under challenging conditions, dietary XOS continue to shape gut microbiota composition, but the effects on diversity were no longer visible, reinforcing the need for future longer-term experiments While our study is highly relevant on exploring the potential of XOS, especially on shaping gut microbiota, further research is warranted to elucidate its role on shrimp health.

## Figures and Tables

**Table 1:** Initial and final weight, relative growth rate (RGR), feed conversion ratio (FCR), feed intake, and survival of whiteleg shrimp (*P. vannamei*) fed the experimental diets (control, XOS_0.1, and XOS_1) for 14 days.

	CTRL	XOS_0.1	XOS_1
Initial weight (g)	0.84 ± 0.01	0.85 ± 0.02	0.85 ± 0.01
Final weight (g)	3.09 ± 0.15	3.08 ± 0.10	3.13 ± 0.17
RGR (% day^−1^)	9.71 ± 0.40	9.65 ± 0.28	9.72 ± 0.38
FCR	0.86 ± 0.06	0.81 ± 0.03	0.83 ± 0.06
Feed intake (% ABW day^−1^)	7.06 ± 0.34	6.64 ± 0.30	6.94 ± 0.31
Survival (%)	96.5 ± 1.7	98.1 ± 1.6	94.2 ± 3.1

Results expressed as mean ± standard deviation. For initial weight, n = 52 experimental units, and for the remaining parameters, n = 6 experimental units.

**Table 2:** Relative gene expression levels of genes from shrimp hepatopancreas under different dietary conditions (control, XOS_0.1, and XOS_1) and between different time points (after the feeding trial—0 h—and after the bacterial challenge—72 h).

Gene Acronym	Group	0 h		72 h			
				Non-Infected		Infected	
		Mean	SEM	Mean	SEM	Mean	SEM
*IAP*	Control	1.00	0.60	0.24	0.17	0.27	0.11
	XOS_0.1	0.20	0.04			0.13	0.07
	XOS_1	1.86	1.69			0.18	0.05
*PEN3*	Control	1.00	0.17	0.71	0.18	1.43	0.33
	XOS_0.1	1.05	0.30			0.84	0.23
	XOS_1	3.05	2.49			1.02	0.22
*LZC*	Control	1.00	0.58	12.50	8.56	5.60	1.35
	XOS_0.1	0.52	0.13			2.24	1.09
	XOS_1	16.42	15.80			3.52	1.80
*TRYP*	Control	1.00	0.25	0.27	0.06	0.55	0.11
	XOS_0.1	0.84	0.21			0.52	0.21
	XOS_1	2.02	0.98			0.44	0.16
*GPX2*	Control	1.00	0.17	0.65	0.07	0.58	0.08
	XOS_0.1	0.75	0.08			0.66	0.09
	XOS_1	0.90	0.14			0.59	0.06
*LECTIN2*	Control	1.00	0.23	0.17	0.03	0.46	0.12
	XOS_0.1	0.45	0.09			0.30	0.12
	XOS_1	0.53	0.11			0.21	0.04
*TRX2*	Control	1.00	0.23	1.10	0.31	0.51	0.10
	XOS_0.1	0.56	0.08			0.53	0.10
	XOS_1	1.05	0.40			0.42	0.05
*HSP70*	Control	1.00	0.38	4.62	3.19	3.39	1.05
	XOS_0.1	0.52	0.13			7.57	6.24
	XOS_1	13.52	13.22			2.15	0.60
*CASP3*	Control	1.00	0.24	0.33	0.08	0.45	0.15
	XOS_0.1	0.52	0.09			0.75	0.27
	XOS_1	1.32	1.04			0.34	0.06
*GST*	Control	1.00	0.81	0.47	0.40	0.01	0.01
	XOS_0.1	0.14	0.11			0.78	0.73
	XOS_1	0.15	0.05			1.52	1.49

Data represent the mean and standard error of the mean (SEM) of independent biological replicates from each group.

**Table 3:** Differently abundant bacterial genera identified in shrimp fed XOS diets (XOS_0.1 and XOS_1) compared to control at 0 and 72 h.

Genus	vs. Control at 0 h		vs. Control at 72 h	
	XOS_0.1	XOS_1	XOS_0.1	XOS_1
*Acinetobacter*	−	−	−/+	−/+
*Anaerococcus*			−	−
*Anaeromyxobacter*			+	
*Aquabacterium*			+	
*Bacillus*		+		
*Bacteroides*			−	−
*Brevundimonas*			+	
*Burkholderia-Caballeronia-Paraburkholderia*			+++	
*Congregibacter*		−	−	
*Corynebacterium*			−/+	
*Corynebacterium_1*			+	−
*Cupriavidus*			−	
*Cutibacterium*			−	−
*Cytophaga*			−	−
*Delftia*				−
*Demequina*	+	+++		
*Devosia*			+	+
*Domibacillus*		+		
*Enhydrobacter*			−	−
*Finegoldia*				−
*Flavobacterium*	+	+	−	−
*Fusibacter*	−	−		
*Gilvimarinus*	+	+		
*Haloferula*		++		
*Hoeflea*		−		
*Hwangdonia*	+	+		
*Ilumatobacter*		+		
*Kocuria*			−	−
*Labrenzia*		++		
*Lawsonella*				−
*Leisingera*	+	+		
*Lentimonas*		−		
*Loktanella*	+			
*Luteolibacter*	+	+		
*Maritalea*		++		
*Marmoricola*			−	
*Massilia*			+	
*Motilimonas*	−	−		
*Muricauda*	+	+++		
*Nautella*		+		
*Oceanobacter*	−	−		
*Owenweeksia*	+	+		
*Palleronia*	+	−		
*Paracoccus*	+	+	+	
*Pelomonas*			−	−
*Peptoniphilus*			−	−
*Pseudomonas*	−/+	−/+	+	
*Qipengyuania*	−	−	−	
*Reyranella*			++	
*Rheinheimera*		+		
*Rhodoglobus*			+	
*Rhodovulum*		+		
*Romboutsia*			+	
*Roseibacillus*		+		
*Roseobacter*		+		
*Roseovarius*	+			
*Ruegeria*	+++	++++		
*Shewanella*			−	−
*Silicimonas*		++		
*Sphingomonas*			−	
*Staphylococcus*			−−	−−
*Streptococcus*			++	
*Tamlana*	+	+		
*Tenacibaculum*	++++	++++		−−
*Terasakiella*	−	−		
*Thermomonas*			−	−
*Thiothrix*				+
*Trichormus_HINDAK_2001–4*	−	−		
*Vibrio*	+	−	+	
*Weissella*				+
*Xanthomarina*	+	+		

Symbols “+” and “−” represent genera that were significantly more (+) and less (−) abundant in the XOS groups compared to the control. The number of times that the symbol (+ or −) appears indicates the number of unique ASVs within that genus that met the threshold: FDR-adjusted *p*-value < 0.05 and fold change >2. To facilitate visualization, more abundant genera are present in green, while less abundant genera are presented in red. Those that are ambiguous are presented in yellow.

**Table 4:** Dietary composition of the experimental diets used to culture juvenile whiteleg shrimp (*P. vannamei*).

Ingredients (%)	CTRL	XOS_0.1	XOS_1
Fishmeal ^1^	10.50	10.50	10.50
Squid liver meal ^2^	2.00	2.00	2.00
Poultry meal ^3^	6.00	6.00	6.00
Corn gluten meal ^4^	3.90	3.90	3.90
Soybean meal ^5^	34.00	34.00	34.00
Wheat meal ^6^	28.40	28.40	28.40
Wheat bran ^7^	7.00	7.00	7.00
Vitamin and mineral premix ^8^	1.00	1.00	1.00
Choline chloride 50% ^9^	0.20	0.20	0.20
Antioxidant ^10^	0.20	0.20	0.20
Sodium propionate ^11^	0.10	0.10	0.10
Monoammonium phosphate ^12^	0.60	0.60	0.60
Calcium carbonate ^13^	0.40	0.40	0.40
Astaxanthin ^14^	0.05	0.05	0.05
Binder ^15^	0.30	0.30	0.30
L-Lysine HCl 99% ^16^	0.15	0.15	0.15
DL-Methionine ^17^	0.20	0.20	0.20
Soy lecithin ^18^	2.00	2.00	2.00
Fish oil ^19^	2.00	2.00	2.00
Soybean oil ^20^	1.00	1.00	1.00
Salicornia XOS ^21^		0.10	1.00

^1^ Super-Prime: 68% CP, 8% CF, Pesquera Diamante, Lima, Peru; ^2^ Squid liver meal: 78% CP, 8% CF, Sopropêche, Wimille, France; ^3^ Poultry meal 65: 65% CP, 12% CF, SAVINOR UTS, Trofa, Portugal; ^4^ Corn gluten meal: 61% CP, 4% CF, COPAM, Lisbon, Portugal; ^5^ Solvent extracted soybean meal: 43% CP, 2.7% CF, CARGILL, Barcelona, Spain; ^6^ Wheat meal: 10.2% CP; 1.2% CF, MOLISUR, Málaga, Spain; ^7^ Wheat bran: 15.8% CP; 4.7% CF, Ribeiro e Sousa Lda., Lisbon, Portugal; ^8^ PREMIX Lda., Viana do Castelo, Portugal: Vitamins (IU or mg/kg diet): DL-alpha tocopherol acetate, 100 mg; sodium menadione bisulphate, 25 mg; retinyl acetate, 20,000 IU; DL-cholecalciferol, 2000 IU; thiamin, 30 mg; riboflavin, 30 mg; pyridoxine, 20 mg; cyanocobalamin, 0.1 mg; nicotinic acid, 200 mg; folic acid, 15 mg; ascorbic acid, 500 mg; inositol, 500 mg; biotin, 3 mg; calcium pantothenate, 100 mg; choline chloride, 1000 mg, betaine, 500 mg, excipient wheat; ^9^ ORFFA, Breda, The Netherlands; ^10^ VERDILOX PX, KEMIN EUROPE NV, Herentals, Belgium; ^11^ PREMIX Lda., Viana do Castelo, Portugal; ^12^ MAP: 26% P, Phosphea, Prahovo, Serbia; ^13^ Calcium carbonate: 40% Ca, PREMIX Lda., Viana do Castelo, Portugal; ^14^ DSM Nutritional Products, Basel, Switzerland; ^15^ PELLETIN, Sappi Southern Africa Ltd., Johannesburg, South Africa; ^16^ L-Lysine HCl 99%: Ajinomoto Eurolysine SAS, Paris, France; ^17^ Rhodimet NP99, ADISSEO, Antony, France; ^18^ LECICO GmbH, Hamburg, Germany; ^19^ Sopropêche, Wimille, France; ^20^ J.C. Coimbra Lda., Setúbal, Portugal; ^21^ CELABOR, Herve, Belgium.

**Table 5:** Proximate composition of the experimental diets used to culture juvenile whiteleg shrimp.

Proximate Composition (% Feed)	CTRL	XOS_0.1	XOS_1
Dry matter	90.6	90.6	90.6
Crude protein	36.1	36.1	36.1
Crude lipids	7.1	7.1	7.1
Ash	6.8	6.8	6.8
Energy (KJ g^−1^ DM)	18.4	18.4	18.4

**Table 6:** Forward (F) and reverse (R) primers used for quantitative PCR in whiteleg shrimp hepatopancreas to assess health and immune condition.

Gene	Acronym	Primer Sequences (5′–3′)	Acc. No.
*Ribossomal protein L8*	*RPL8*	F: AGCCAAGCAAGATGGGTCG	XM_027355167.1
		R: TGTAACGATAAGGGTCACGGAAG	
*Glyceraldehyde 3-phosphate dehydrogenase*	*GADPH*	F: AAAGGTAGGAATTGCCCCCG	XM_027372388.1
		R: AGGGATGAGACTAGCACGACT	
*Inhibitor of opoptosis protein*	*IAP*	F: CAACACCTGCCTCAGGACAA	GQ293142.1
		R: CTTCCATTGCCTCCTCGTCT	
*Penaeidin 3*	*PEN3*	F:ATACCCAGGCCACCACCCTT	XM_027360479.1
		R: TGACAGCAACGCCCTAACC	
*Lysozyme C-like*	*LZC*	F: CGGGAAAGGCTATTCTGCCT	XM_027352840.1
		R: CCAGCACTCTGCCATGTACT	
*Trypsin*	*TRYP*	F: CGGAGAGCTGCCTTACCAG	XM_027367621.1
		R: TCGGGGTTGTTCATGTCCTC	
*Glutathione peroxidase 2-like*	*GPX2*	F: AGGGACTTCCACCAGATG	XM_027372127.1
		R: CAACAACTCCCCTTCGGTA	
*C-type lectin 2-like*	*LECTIN2*	F: GCTTCTGTTGGTGCTGTTGGC	DQ858899.2
		R: GTTCCCTTCCCGTATGTGGC	
*Thioredoxin 2*	*TRX2*	F: TTCCTGAAGGTGGATGTGGA	XM_027377405.1
		R: AGTTGGCACCAGACAAGCTG	
*Heath shock protein 70*	*HSP70*	F: CAACGATTCTCAGCGTCAGG	XM_027369405.1
		R: ACCTTCTTGTCGAGGCCGTA	
*Caspase 3*	*Casp3*	F: ACATTTCTGGGCGGAACACC	KC660103.1
		R: GTGACACCCGTGCTTGTACA	
*Glutathione S-transferase*	*GST*	F: CACCTACGAACACTACGAAC	XM_027351980.1
		R: GGTTCTTGAAGCCGTCGAG	
*Rod shape-determining gene*, *subunit B*	*mreB*	F: TGAAGCTGTGATCAACTACG	D0XAE4_VIBH1
		R: CCTGACAGTGGCTCTTGTAA	
*Transmembrane transcriptor regulator*	*toxR*	F: GAAGCAGCACTCACCGAT	AY247418
		R: GGTGAAGACTCATCAGCA	
*Topoisomerase I*	*topA*	F: TGGCGCAGCGTCTATACG	JF930499
		R: TATTTGTCACCGAACTCAGAACC	

## Data Availability

The original contributions presented in this study are included in the article/Supplementary Materials. Further inquiries can be directed to the corresponding author.

## Supplementary Materials

The following supporting information can be downloaded at: https://www.mdpi.com/article/10.3390/ijms262411978/s1.

## References

[1] (2022). The State of World Fisheries and Aquaculture 2022.

[2] Villarreal H. (2023). Shrimp farming advances, challenges, and opportunities. J. World Aquac. Soc..

[3] Ma S., Kim A., Lee W., Kim S., Lee S., Yoon D., Bae J.S., Park C., Kim S. (2020). Vibrio harveyi infection significantly alters amino acid and carbohydrate metabolism in whiteleg shrimp, litopenaeus vannamei. Metabolites.

[4] Amatul-Samahah M.A., Wan Omar W.H.H., Mohd Ikhsan N.F., Amal Azmai M.N., Zamri-Saad M., Ina-Salwany M.Y. (2020). Vaccination trials against vibriosis in shrimp: A review. Aquac. Rep..

[5] Garibay-Valdez E., Martínez-Córdova L.R., López-Torres M.A., Almendariz-Tapia F.J., Martínez-Porchas M., Calderón K. (2020). The implication of metabolically active *Vibrio* spp. in the digestive tract of Litopenaeus vannamei for its post-larval development. Sci. Rep..

[6] Angthong P., Uengwetwanit T., Uawisetwathana U., Koehorst J.J., Arayamethakorn S., Schaap P.J., Dos Santos V.M., Phromson M., Karoonuthaisiri N., Chaiyapechara S. (2023). Investigating host-gut microbial relationship in Penaeus monodon upon exposure to Vibrio harveyi. Aquaculture.

[7] Gufe C., Merrifield D.L., Hoseinifar S.H., Rattanarojpong T., Khunrae P., Abdel-Tawwab M. (2023). Prebiotic effects of dietary xylooligosaccharides on fish gut microbiota, growth, and immunological parameters—A review. Ann. Anim. Sci..

[8] Gyawali R., Nwamaioha N., Fiagbor R., Zimmerman T., Newman R.H., Ibrahim S.A. (2019). The Role of Prebiotics in Disease Prevention and Health Promotion. Dietary Interventions in Gastrointestinal Diseases.

[9] Lin S.H., Chou L.M., Chien Y.W., Chang J.S., Lin C.I. (2016). Prebiotic Effects of Xylooligosaccharides on the Improvement of Microbiota Balance in Human Subjects. Gastroenterol. Res. Pract..

[10] Gibson G.R., Probert H.M., Van Loo J., Rastall R.A., Roberfroid M.B. (2004). Dietary modulation of the human colonic microbiota: Updating the concept of prebiotics. Nutr. Res. Rev..

[11] Kaur A.P., Bhardwaj S., Dhanjal D.S., Nepovimova E., Cruz-martins N., Kuča K., Chopra C., Singh R., Kumar H., Șen F. (2021). Plant prebiotics and their role in the amelioration of diseases. Biomolecules.

[12] Ma Y., Wu X., Giovanni V., Meng X. (2017). Effects of soybean oligosaccharides on intestinal microbial communities and immune modulation in mice. Saudi J. Biol. Sci..

[13] Neyrinck A.M., Possemiers S., Druart C., van de Wiele T., de Backer F., Cani P.D., Larondelle Y., Delzenne N.M. (2011). Prebiotic effects of wheat Arabinoxylan related to the increase in bifidobacteria, roseburia and bacteroides/prevotella in diet-induced obese mice. PLoS ONE.

[14] Aachary A.A., Prapulla S.G. (2011). Xylooligosaccharides (XOS) as an Emerging Prebiotic: Microbial Synthesis, Utilization, Structural Characterization, Bioactive Properties, and Applications. Compr. Rev. Food Sci. Food Saf..

[15] Gibson G.R., Hutkins R., Sanders M.E., Prescott S.L., Reimer R.A., Salminen S.J., Scott K., Stanton C., Swanson K.S., Cani P.D. (2017). Expert consensus document: The International Scientific Association for Probiotics and Prebiotics (ISAPP) consensus statement on the definition and scope of prebiotics. Nat. Rev. Gastroenterol. Hepatol..

[16] Li F., Li Q., Zhang Y., Zhou X., Yi R., Zhao X. (2021). Effects of Xylooligosaccharides on Lipid Metabolism, Inflammation, and Gut Microbiota in C57BL/6J Mice Fed a High-Fat Diet. Front. Pharmacol..

[17] Lyu Y., Debevere S., Bourgeois H., Ran M., Broeckx B.J.G., Vanhaecke L., Van de Wiele T., Hesta M. (2020). Dose-Dependent Effects of Dietary Xylooligosaccharides Supplementation on Microbiota, Fermentation and Metabolism in Healthy Adult Cats. Molecules.

[18] Broekaert W.F., Courtin C.M., Verbeke K., van de Wiele T., Verstraete W., Delcour J.A. (2011). Prebiotic and other health-related effects of cereal-derived arabinoxylans, arabinoxylan-oligosaccharides, and xylooligosaccharides. Crit. Rev. Food Sci. Nutr..

[19] Chen Y., Xie Y., Ajuwon K.M., Zhong R., Li T., Chen L., Zhang H., Beckers Y., Everaert N. (2021). Xylo-Oligosaccharides, Preparation and Application to Human and Animal Health: A Review. Front. Nutr..

[20] (2020). Global Forest Resources Assessment 2020.

[21] Hameed A., Hussain S., Rasheed A., Ahmed M.Z., Abbas S. (2024). Exploring the Potentials of Halophytes in Addressing Climate Change-Related Issues: A Synthesis of Their Biological, Environmental, and Socioeconomic Aspects. World.

[22] Lima A.R., Cristofoli N.L., Rosa da Costa A.M., Saraiva J.A., Vieira M.C. (2023). Comparative study of the production of cellulose nanofibers from agro-industrial waste streams of Salicornia ramosissima by acid and enzymatic treatment. Food Bioprod. Process..

[23] Monção M., Thoresen P.P., Wretborn T., Lange H., Rova U., Christakopoulos P., Matsakas L. (2023). A novel biorefinery concept based on marginally used halophyte biomass. Sustain. Energy Fuels.

[24] Álvarez-Rodríguez M., Pereiro P., Reyes-López F.E., Tort L., Figueras A., Novoa B. (2018). Analysis of the long-lived responses induced by immunostimulants and their effects on a viral infection in Zebrafish (*Danio rerio*). Front. Immunol..

[25] Sun Y., Wang G., Peng K., Huang Y., Cao J., Huang W., Chen B., Hu J. (2019). Effects of dietary xylooligosaccharides on growth performance, immunity and Vibrio alginolyticus resistance of juvenile Litopenaeus vannamei. Aquac. Res..

[26] Kazuń B., Kazuń K., Małaczewska J., Kamiński R., Adamek-Urbańska D., Sikorska J., Wolnicki J., Szudrowicz H. (2023). Effects of long-term administration of various dietary prebiotic supplements on the growth, immune cell activity and digestive tract histology of juvenile vimba (Vimba vimba). J. Vet. Res..

[27] Yuan Y., Jin M., Fang F., Tocher D.R., Betancor M.B., Jiao L., Hong Y., Zhou Q. (2022). New Insight Into the Molting and Growth in Crustaceans: Regulation of Energy Homeostasis Through the Lipid Nutrition. Front. Mar. Sci..

[28] Li S., Li W., Chen F., Zhu X., Chen H.Y., Hao H., Wang K.J. (2023). Metabolomic and transcriptomic analysis reveals immune and hormone modulation at the molting stage of juvenile mud crabs challenged with Staphylococcus aureus and Vibrio alginolyticus. Aquaculture.

[29] Gibson R., Barker P.L. (1979). The decapod hepatopancreas. Oceanogr. Mar. Biol..

[30] Yan F., Tian S., Chen H., Gao S., Dong X., Du K. (2022). Advances in xylooligosaccharides from grain byproducts: Extraction and prebiotic effects. Grain Oil Sci. Technol..

[31] Callac N., Giraud C., Boulo V., Wabete N., Pham D. (2023). Microbial biomarker detection in shrimp larvae rearing water as putative bio-surveillance proxies in shrimp aquaculture. PeerJ.

[32] Dong P., Guo H., Huang L., Zhang D., Wang K. (2023). Glucose addition improves the culture performance of Pacific white shrimp by regulating the assembly of Rhodobacteraceae taxa in gut bacterial community. Aquaculture.

[33] Guo H., Fu X., He J., Wang R., Yan M., Wang J., Dong P., Huang L., Zhang D. (2023). Gut bacterial consortium enriched in a biofloc system protects shrimp against Vibrio parahaemolyticus infection. Microbiome.

[34] Zhou Z., Wen M., Xiang L., Shen H., Jiang G., Cheng J., Hu Y., Qian J. (2024). Segmental variations in intestinal microbiota composition and functional capacity along the digestive tract of Litopenaeus vannamei. Aquac. Rep..

[35] Huang L., Guo H., Liu Z., Chen C., Wang K., Huang X., Chen W., Zhu Y., Yan M., Zhang D. (2022). Contrasting patterns of bacterial communities in the rearing water and gut of Penaeus vannamei in response to exogenous glucose addition. Mar. Life Sci. Technol..

[36] Fu X., He J., Wang J., Shen F., Qiu J., Chen C., Zhang D., Guo H. (2024). Specific gut bacterial taxa inhabited in healthy shrimp (*Penaeus vannamei*) confer protection against Vibrio parahaemolyticus challenge. Aquaculture.

[37] Alvarez-Ruiz S.A.P., Luna-González A., Escamilla-Montes R., Fierro-Coronado A., Diarte-Plata G., García-Gutiérrez C., Peraza-Gómez V. (2022). Gut bacterial profile associated with healthy and diseased (AHPND) shrimp Penaeus vannamei. Lat. Am. J. Aquat. Res..

[38] Qi C., Wang L., Liu M., Jiang K., Wang M., Zhao W., Wang B. (2017). Transcriptomic and morphological analyses of Litopenaeus vannamei intestinal barrier in response to Vibrio paraheamolyticus infection reveals immune response signatures and structural disruption. Fish Shellfish Immunol..

[39] Duan Y., Wang Y., Huang J., Li H., Dong H., Zhang J. (2021). Toxic effects of cadmium and lead exposure on intestinal histology, oxidative stress response, and microbial community of Pacific white shrimp Litopenaeus vannamei. Mar. Pollut. Bull..

[40] Reyes G., Andrade B., Betancourt I., Panchana F., Preciado C., Bayot B. (2024). Bacterial communities and signatures in the stomach and intestine of juvenile Penaeus (litopenaeus) vannamei shrimp affected by acute hepatopancreatic necrosis disease. Heliyon.

[41] Liang X., Luo X., Lin H., Han F., Qin J.G., Chen L., Xu C., Li E. (2022). Growth, Health, and Gut Microbiota of Female Pacific White Shrimp, Litopenaeus vannamei Broodstock Fed Different Phospholipid Sources. Antioxidants.

[42] Huang X., Gu Y., Zhou H., Xu L., Cao H., Gai C. (2020). Acinetobacter venetianus, a potential pathogen of red leg disease in freshwater-cultured whiteleg shrimp Penaeus vannamei. Aquac. Rep..

[43] Yasin A., Begum K., Eshik M.E., Punom N.J., Ahmmed S., Rahman M.S. (2022). Molecular identification and antibiotic resistance patterns of diverse bacteria associated with shrimp PL nurseries of Bangladesh: Suspecting Acinetobacter venetianus as future threat. PeerJ.

[44] Kuebutornye F.K.A., Abarike E.D., Lu Y. (2019). A review on the application of Bacillus as probiotics in aquaculture. Fish Shellfish Immunol..

[45] Proespraiwong P., Mavichak R., Imaizumi K., Hirono I., Unajak S. (2023). Evaluation of *Bacillus* spp. as Potent Probiotics with Reduction in AHPND-Related Mortality and Facilitating Growth Performance of Pacific White Shrimp (*Litopenaeus vannamei*) Farms. Microorganisms.

[46] Kanjan P., Kimtun A., Chaimongkol S., Sakpetch P. (2022). Probiotic Weissella cibaria KY10 derived from digestive tract of healthy shrimp exhibits strong antibacterial effects against Vibrio parahaemolyticus causing AHPND in shrimp. Aquac. Res..

[47] Philippot L., Griffiths B.S., Langenheder S. (2021). Microbial Community Resilience across Ecosystems and Multiple Disturbances. Microbiol. Mol. Biol. Rev..

[48] Nicholson J.K., Holmes E., Kinross J., Burcelin R., Gibson G., Jia W., Pettersson S. (2012). Metabolic Interactions. Science.

[49] Legrand T.P.R.A., Catalano S.R., Wos-Oxley M.L., Stephens F., Landos M., Bansemer M.S., Stone D.A.J., Qin J.G., Oxley A.P.A. (2018). The inner workings of the outer surface: Skin and gill microbiota as indicators of changing gut health in Yellowtail Kingfish. Front. Microbiol..

[50] Zeng S., He J., Huang Z. (2024). The intestine microbiota of shrimp and its impact on cultivation. Appl. Microbiol. Biotechnol..

[51] Xiong J., Zhu J., Dai W., Dong C., Qiu Q., Li C. (2017). Integrating Gut Microbiota Immaturity and Disease-Discriminatory Taxa to Diagnose the Initiation and Severity of Shrimp Disease. Environ. Microbiol..

[52] Cornejo-Granados F., Lopez-Zavala A.A., Gallardo-Becerra L., Mendoza-Vargas A., Sánchez F., Vichido R., Brieba L.G., Viana M.T., Sotelo-Mundo R.R., Ochoa-Leyva A. (2017). Microbiome of Pacific Whiteleg shrimp reveals differential bacterial community composition between Wild, Aquacultured and AHPND/EMS outbreak conditions. Sci. Rep..

[53] Chang Y.-T., Ko H.-T., Wu P.-L., Kumar R., Wang H.-C., Lu H.-P. (2023). Gut microbiota of Pacific white shrimp (*Litopenaeus vannamei*) exhibits distinct responses to pathogenic and non-pathogenic Vibrio parahaemolyticus. Microbiol. Spectr..

[54] Tran P.T.N., Kumar V., Bossier P. (2020). Do acute hepatopancreatic necrosis disease-causing PirABVP toxins aggravate vibriosis?. Emerg. Microbes Infect..

[55] Rosas C., Cuzon G., Gaxiola G., Le Priol Y., Pascual C., Rossignyol J., Contreras F., Sanchez A., Van Wormhoudt A. (2001). Metabolism and growth of juveniles of Litopenaeus vannamei: Effect of salinity and dietary carbohydrate levels. J. Exp. Mar. Bio. Ecol..

[56] Lodish H., Berk A., Kaiser C., Krieger M., Bretscher A., Ploegh H., Martin K., Yaffe M., Amon A. (2021). Molecular Cell Biology.

[57] Den Besten G., Van Eunen K., Groen A.K., Venema K., Reijngoud D.J., Bakker B.M. (2013). The role of short-chain fatty acids in the interplay between diet, gut microbiota, and host energy metabolism. J. Lipid Res..

[58] Xu J., Gordon J.I. (2003). Honor thy symbionts. Proc. Natl. Acad. Sci. USA.

[59] Herigstad B., Hamilton M., Heersink J. (2001). How to optimize the drop plate method for enumerating bacteria. J. Microbiol. Methods.

[60] Shankregowda A.M., Siriyappagouder P., Kuizenga M., Bal T.M.P., Abdelhafiz Y., Eizaguirre C., Fernandes J.M.O., Kiron V., Raeymaekers J.A.M. (2023). Host habitat rather than evolutionary history explains gut microbiome diversity in sympatric stickleback species. Front. Microbiol..

[61] Müller T., Kalxdorf M., Longuespée R., Kazdal D.N., Stenzinger A., Krijgsveld J. (2020). Automated sample preparation with SP 3 for low-input clinical proteomics. Mol. Syst. Biol..

[62] Powell D.R. (2019). Degust: Interactive RNA-seq analysis. Zenodo.

[63] Huang D.W., Sherman B.T., Lempicki R.A. (2009). Bioinformatics enrichment tools: Paths toward the comprehensive functional analysis of large gene lists. Nucleic Acids Res..

[64] Szklarczyk D., Kirsch R., Koutrouli M., Nastou K., Mehryary F., Hachilif R., Gable A.L., Fang T., Doncheva N.T., Pyysalo S. (2023). The STRING database in 2023: Protein–protein association networks and functional enrichment analyses for any sequenced genome of interest. Nucleic Acids Res..

